# Genome-wide analysis of plant specific *YABBY* transcription factor gene family in carrot (*Dacus carota*) and its comparison with Arabidopsis

**DOI:** 10.1186/s12863-024-01210-4

**Published:** 2024-03-05

**Authors:** Mujahid Hussain, Muhammad Mubashar Javed, Adnan Sami, Muhammad Shafiq, Qurban Ali, Hafiz Sabah-Ud-Din Mazhar, Javaria Tabassum, Muhammad Arshad Javed, Muhammad Zeeshan Haider, Muhammad Hussain, Irfan Ali Sabir, Daoud Ali

**Affiliations:** 1 Department of Horticulture, Faculty of Agriculture Sciences, University of the Punjab, Lahore P. O BOX, Lahore, 54590 Pakistan; 2 Department of Plant Breeding & Genetics, Faculty of Agriculture Sciences, University of the Punjab, P.O BOX, Lahore, 54590 Pakistan; 3 College of Horticulture, South China Agricultural University, Guangzhou, 510642 China; 4 Department of Zoology, College of Science, King Saud University, PO Box 2455, Riyadh, 11451 Saudi Arabia

**Keywords:** Bioinformatics, Carrot, Gene family, Genomic analysis, Specific plant transcription factors, *YABBY*

## Abstract

*YABBY* gene family is a plant-specific transcription factor with DNA binding domain involved in various functions i.e. regulation of style, length of flowers, and polarity development of lateral organs in flowering plants. Computational methods were utilized to identify members of the *YABBY* gene family, with Carrot (*Daucus carota*) ‘s genome as a foundational reference. The structure of genes, location of the chromosomes, protein motifs and phylogenetic investigation, syntony and transcriptomic analysis, and miRNA targets were analyzed to unmask the hidden structural and functional characteristics *YABBY* gene family in *Carrots*. In the following research, it has been concluded that 11 specific *YABBY* genes irregularly dispersed on all 9 chromosomes and proteins assembled into five subgroups i.e. *AtINO*, *AtCRC*, *AtYAB5*, *AtAFO*, and *AtYAB2,* which were created on the well-known classification of *Arabidopsis*. The wide ranges of *YABBY* genes in carrots were dispersed due to segmental duplication, which was detected as prevalent when equated to tandem duplication. Transcriptomic analysis showed that one of the *DcYABBY* genes was highly expressed during anthocyanin pigmentation in carrot taproots. The cis-regulatory elements (CREs) analysis unveiled elements that particularly respond to light, cell cycle regulation, drought induce ability, ABA hormone, seed, and meristem expression. Furthermore, a relative study among Carrot and Arabidopsis genes of the *YABBY* family indicated 5 sub-families sharing common characteristics. The comprehensive evaluation of YABBY genes in the genome provides a direction for the cloning and understanding of their functional properties in carrots. Our investigations revealed genome-wide distribution and role of *YABBY* genes in the carrots with best-fit comparison to *Arabidopsis thaliana.*

**Supplementary Information:**

The online version contains supplementary material available at 10.1186/s12863-024-01210-4.

## Introduction

*YABBY* plant-specific transcription factors (PSTFs) gene family plays an important role in the development of plants i.e. regulation of style length in flowering plants [1] resistance against abiotic stresses [2], polarity development in plant’s lateral organs [3] developmental processes of vegetative and reproductive organs [4], initiating signals responsible for plant hormonal reactions [5] development of vascular organs [6] development of nectary [7] and germination of seed and processes after germination [8, 9]. The *DcYABBY* genes are members of the *YABBY* superfamily having functionally important domains i.e., Hmg_box and Hmg_box2. These two domains and the YABBY domain contain highly conserved amino acid residues that function in specific DNA binding [10].

The Carrot (*D. carota L*.) is a vital biennial vegetable in Apiaceae family. The family Apiaceae also possess several members i.e. Fennel (*F. vulgare*), celery (*A. graveolens*), parsley (*P. crispum*), cilantro (*C. sativum*) and dill (*A. graveolens*) [11, 12]. Carrot is a cool-season biennial crop used for domestic, commercial, and medicinal purposes initially and cultivated for over 2000 years. It contains sufficient vitamins and amino acids and helps improve eyesight, lowering cholesterol and improving digestion [13, 14]. Antioxidants like carotenoids & phenolic compounds are found in sufficient amounts in carrot, which are beneficial in several biological processes of the human body [15]. While the amount of carotenoids differs noticeably between different genotypes of carrots, which could be due to the physiological and evolutionary distribution of genomics features [16, 17]. Carrots comprise phenolic components with only one aromatic ring (phenolic acids), 3-O-caffeoylquinic [18]. For new marketable carrot varieties, sweetness was considered a significant factor for acceptance [19]. There is a need to develop highly productive varieties of crops like carrot containing richer nutritional value to enhance the production of healthful foods across the globe [20]. For a balanced, secure, and healthy diet, these foods must be accessible worldwide [21]. Carrot faces several physiological damages due to drought [22, 23]. Therefore, we will also try to find out whether the *YABBY* transcription factor gene family can solve this problem.

The research aims to discover and describe the genes belonging to the *YABBY* PSTrFs gene family in the carrot genome using various bioinformatics tools [24]. Concisely, an efficient approach was followed to find the *YABBY* genes family in carrots. This study unveiled *YABBY* genes, revealing their chromosomal locations, exon structures, and the presence of cis-regulatory elements, along with conserved domains.. Broad genome-wide assessment of *YABBY* PSTrF gene family in carrot provides insights to unhide the functional and structural properties which can be used to strengthen the nutritional and food value of other horticulture crops.

## Materials and methods

### Database search and sequence retrieval

It has been confirmed that the experimental data collection complied with relevant institutional, national, and international guidelines and legislation with appropriate permissions from authorities of the Department of Horticulture, University of the Punjab, Lahore, Lahore 54,300, Pakistan. The amino acid sequences of Plant-Specific Transcription Factors (PSTrFs), specifically YABBY, were obtained from the peptide genome of *Arabidopsis thaliana* through the Pfam database (Gene ID: PF04690). The YABBY gene’s 164 amino acid sequences were separated from the *Arabidopsis thaliana* (Accession No. A0A1P8APE2). The following sequences were used in BLAST-P (Basic local protein alignment search tool) for heuristic search against carrot genome using the proteome database at Ensembl plants (https://plants.ensembl.org/index.html) [25–27]. The information on gene IDs, chromosomal position, and sequences of genes and proteins were retrieved. *DcYABBY* amino acid sequences subjected to motif finder (https://www.genome.jp/tools/motif/) [28, 29] and Conserved Domain Database (CDD) (https://www.ncbi.nlm.nih.gov) National Centre for Biotechnology Information (NCBI) (https://www.ncbi.nlm.nih.gov) [30, 31] with customized parameters. The protein sequences that lack in the conserved domain of *YABBY* proteins were diminished from subjective investigations.

### Investigation of physio-chemical characteristics of *DcYABBY* proteins

The properties of *YABBY* proteins i.e. length, molecular weight, and theoretical isoelectric point (pI) were predicted using the ProtParam webserver (https://web.expasy.org) [32, 33]. The subcellular localization of the *DcYABBY* genes was predicted using WoLFPSORT (https://wolfpsort.hgc.jp) [34].

### Gene structure analysis

To predict the genomic architecture of carrot *YABBY* genes, CDS and genomic sequences of *DcYABBY* genes retrieved from Ensembl plants [26, 27]. These sequences and the Newick format of the carrot phylogenetic tree were subjected to a Gene Structure Display Server (GSDS)(http://gsds.gao-lab.org) [35].

### Duplication and syntenic gene analysis

The alignment of protein sequences was conducted using Molecular Evolutionary Genetic Analysis (MEGA) with default parameters. The ratio between the Ka and Ks was predicted using TB tools, and genetic divergence time was calculated using the eq. T = Ks/2r. The “r” signifies a neutral substitution rate (5.2 × 10^−9^ substitutions per site per year) [36, 37].

Duplication events of *DcYABBY* genes were checked with the Multiple Collinearity Scan toolkit (MCScanX) with default settings [38, 39]. Dual synteny analysis of carrot was performed with three crops i.e. Arabidopsis, cucumber, and musk melon. A synteny graph of paralogous of *DcYABBY* genes was created with circos module using TB tools [40].

### Transcriptomic analysis

To check the specific expressions of *DcYABBY* genes RNA-Seq data was downloaded from NCBI Geo (https://www.ncbi.nlm.nih.gov › geo) [41–43]. A log2 transformation was created to check genes’ expression levels in the Reads per Kilo Base per Million (RPKM) values for different *DcYABBY* genes. Using TB tool, a heat map was generated to display the expression level of the different genes [40, 44, 45].

### Analysis of microRNA target sites

The PmiREN webserver (https://academic.oup.com) was utilized to acquire mature miRNA sequences for carrot species [46, 47]. To identify micro-RNAs targeting DcYABBY genes in carrots, the CDS sequences of DcYABBY genes were inputted into the miRNA and target section of the psRNA Target website (https://bio.tools/psrnatarget). Subsequently, the corresponding complementary miRNAs and their targets were retrieved from this analysis [48, 49].

## Results

### Identification of the *YABBY* genes in carrot

**Fig. 1:**
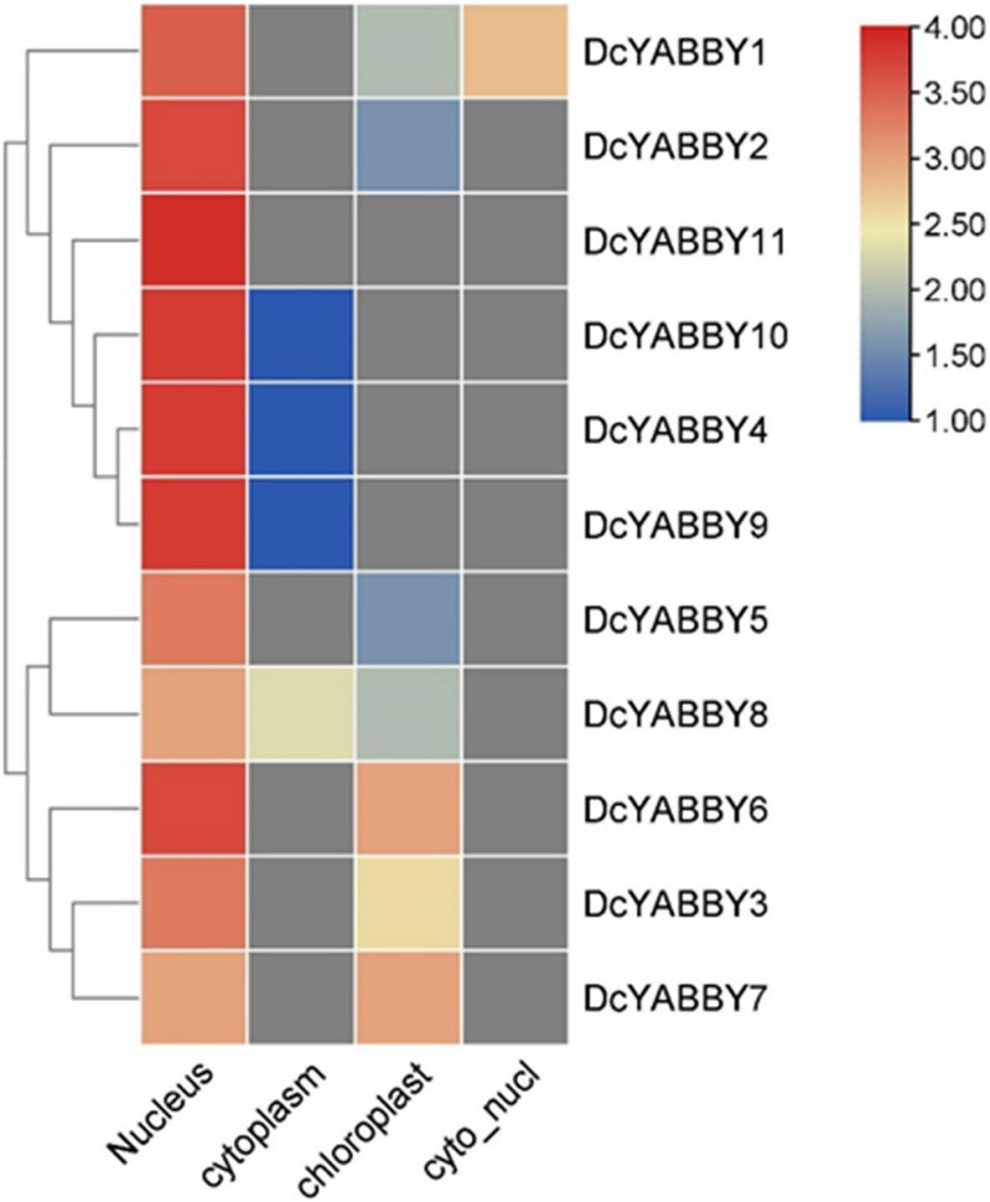
Heat Map representing Sub-cellular localization of all 11 *DcYABBY* genes to various regions of the plant cell including nucleus, cytoplasm and chloroplast. Grey colour represents absence of respective gene in specific region, white colour is showing minimum functional presence of corresponding gene and Red colour represent maximum value of functionally important gene in that particular region

In total 22 *DcYABBY* proteins were identified from proteomic blasts in the carrot genome, and complete domain-possessing sequences were subjected to further investigations. Total 11 sequences of *DcYABBY* genes were selected for analysis. The range of amino acid length of *DcYABBY* genes was between 105 and 229 amino acids, while molecular weight was between 12.17 and 25.23 kDa. The *DcYABBY8* is the shortest, and *DcYABBY1* is the longest protein (Table 1). The pI value of the recognized proteins was extended from 6.82 to 9.16, and it might be due to the increasing number of hydrophobic amino acids. Subcellular localization of these 11 *YABBY* genes depicted that most of these genes were localized towards the nucleus, including a few to chloroplast and the least in the cytoplasm, as shown in the Fig. 1.

**Table 1:** Details of 11 non-redundant *YABBY* genes identified from the genome of Carrot

Gene	Accession	Chr	Chromosome Location	Strand	AA	pI	MW
*DcYABBY1*	DCAR_004921	2	1,743,987–1,745,265	+	229	7.71	25.08531
*DcYABBY2*	DCAR_008543	2	42,935,336–42,936,404	–	227	8.13	25.1555
*DcYABBY3*	DCAR_027801	8	19,070,569–19,071,054	+	228	7.71	21.29318
*DcYABBY4*	DCAR_031517	7	23,365,804–23,367,132	+	192	8.62	23.66181
*DcYABBY5*	DCAR_014892	4	17,476,581–17,477,177	+	209	8.99	25.3846
*DcYABBY6*	DCAR_008464	2	42,290,491–42,292,134	+	231	7.71	17.73905
*DcYABBY7*	DCAR_012254	3	45,104,893–45,106,507	–	155	9.33	12.17168
*DcYABBY8*	DCAR_006190	2	22,993,021–22,993,857	–	105	9.22	24.39574
*DcYABBY9*	DCAR_007074	2	31,254,793–31,255,348	+	219	6.82	17.87828
*DcYABBY10*	DCAR_030050	9	19,958,066–19,958,530	+	166	9.16	25.23959
*DcYABBY11*	DCAR_026683	8	29,737,553–29,738,146	–	229	7.71	25.23959

*AA* Amino acid: *MW* Molecular weight: *PI* Isoelectric point: *Chr* Chromosome

### Gene architecture and conserved motifs analysis

**Fig. 2:**
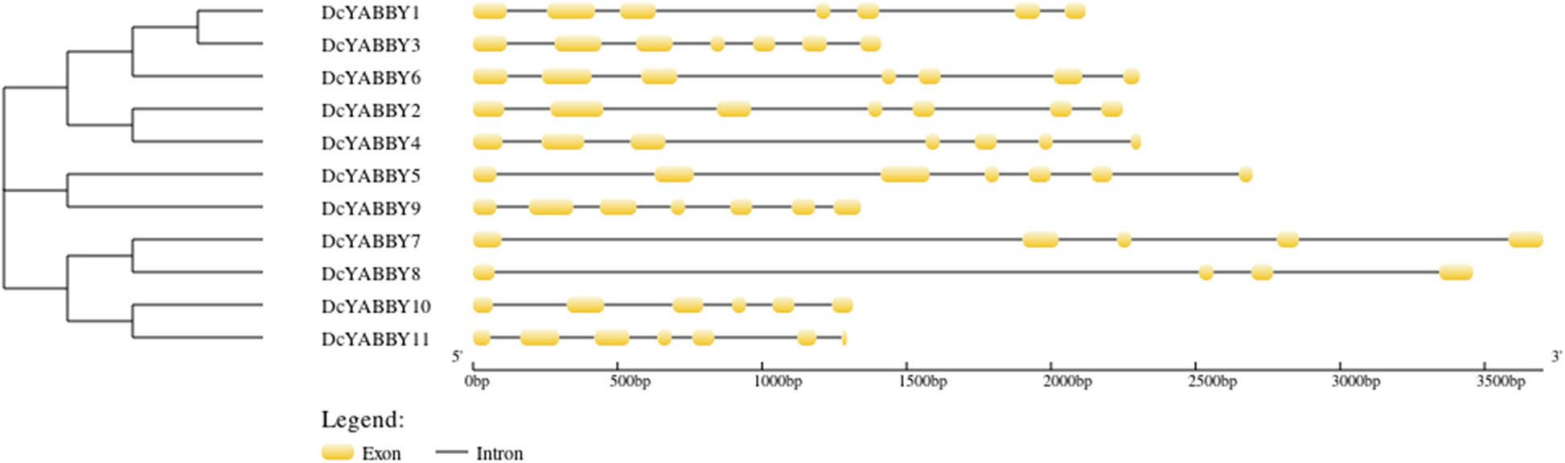
The phylogenic representation of intron-exon structure, showing most of large size gene has less number of coding sequences and vice versa. Meanwhile number of introns and exons are conserved throughout the *YABBY* gene family

**Fig. 3:**
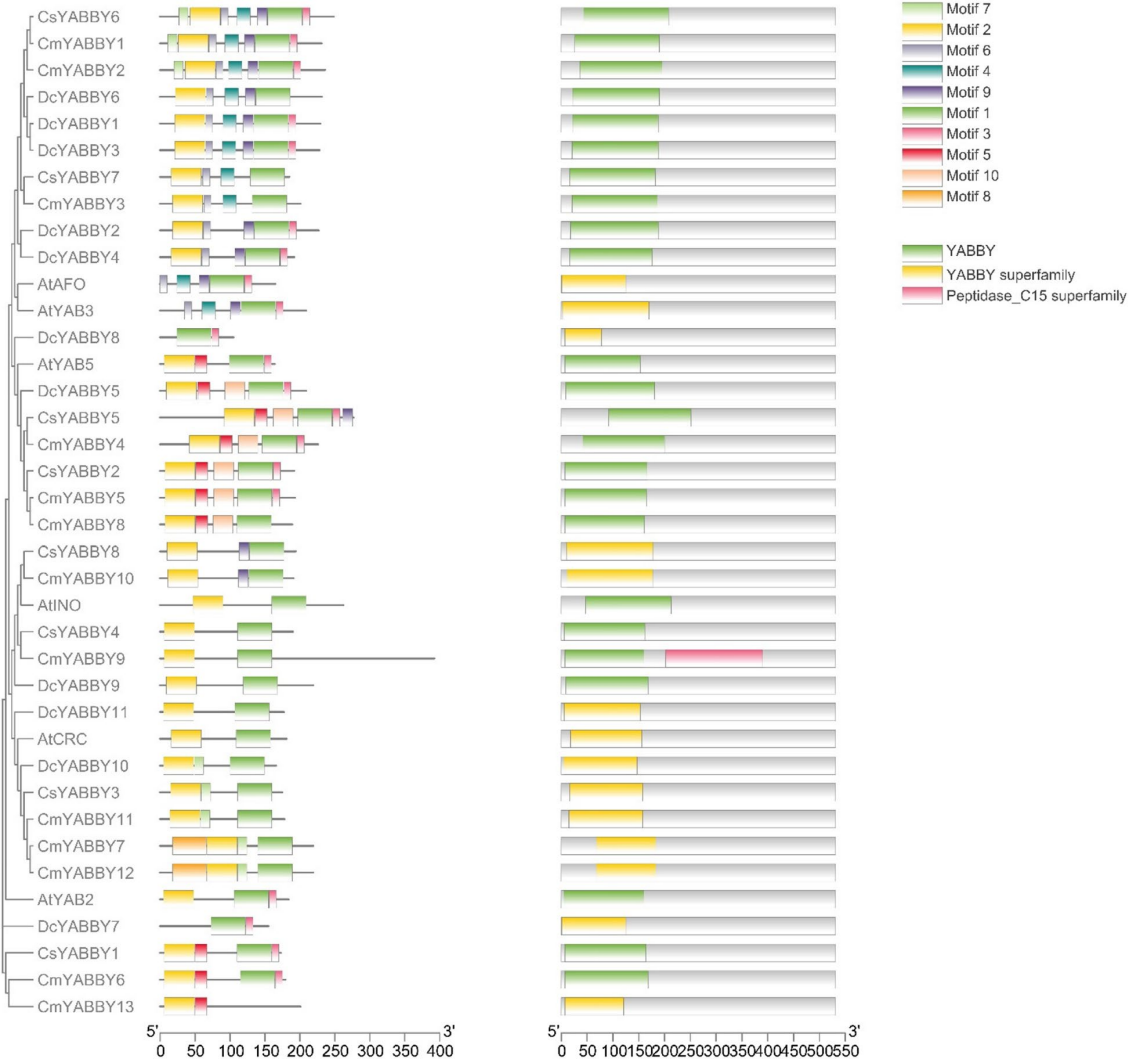
The distribution of 10 motifs along the 11 *YABBY* proteins family in carrot. Motifs is conserved throughout the *YABBY* protein family and are basic structural and functionally important regulator during transient interaction and activation of transcription factors

Seven out of eleven genes comprised 7 exons and 6 introns, while two genes contained 6 exons and 5 introns, and one gene comprised 4 exons and 3 introns & the last gene contained 3 exons and 2 introns (Table S5, Fig. 2). The following coincidence and consistency in several introns and exons leads to the clue that these genes share common ancestors and structural and functional features. The genomic architecture showed that *DcYABBY8* contained 3 introns (27.27%), *DcYABBY7* contains 4 introns (36.36%), *and DcYABBY10* have 5 introns (45.45%) while *DcYABBY1*, *DcYABBY2*, *DcYABBY3*, *DcYABBY4*, *DcYABBY5*, *DcYABBY6*, *DcYABBY9* and *DcYABBY11* contained 6 introns (54.54%) as shown in Fig. 2. There were elucidation and identification of 10 conserved motifs in 11 *DcYABBY* proteins by the motif identification. The *YABBY* domain was conserved in all the *DcYABBY* proteins with several mutations. The motif structure arrangement of the *YABBY* proteins of Group *AtAFO* was conserved, and Motif 2, Motif 3, Motif 5, Motif 6, and Motif 14 were structurally conserved. While *AtCRC* and *AtYAB5* have slight variations, *AtYAB2* is a much-differentiated family member with eight motifs (Table S3, Fig. 3).

### Phylogenetic analysis

**Fig. 4:**
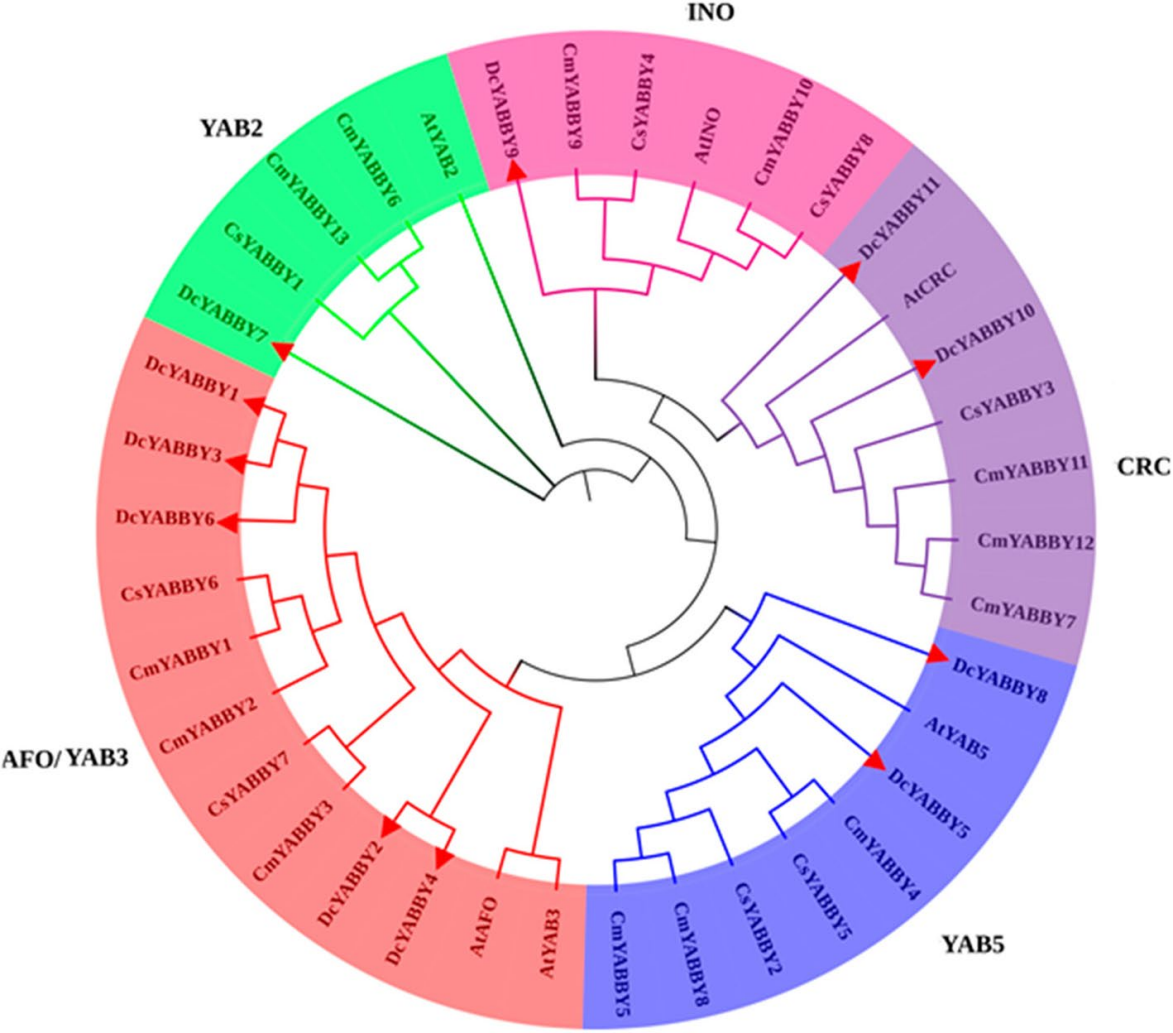
Phylogenetic relationship of 37 *YABBY* genes from four different species i.e. *D. carota*, *A. thaliana*, *C. sativus*, and *C. maxima*. *DcYABBY* genes are represented with red triangle

**Fig. 5:**
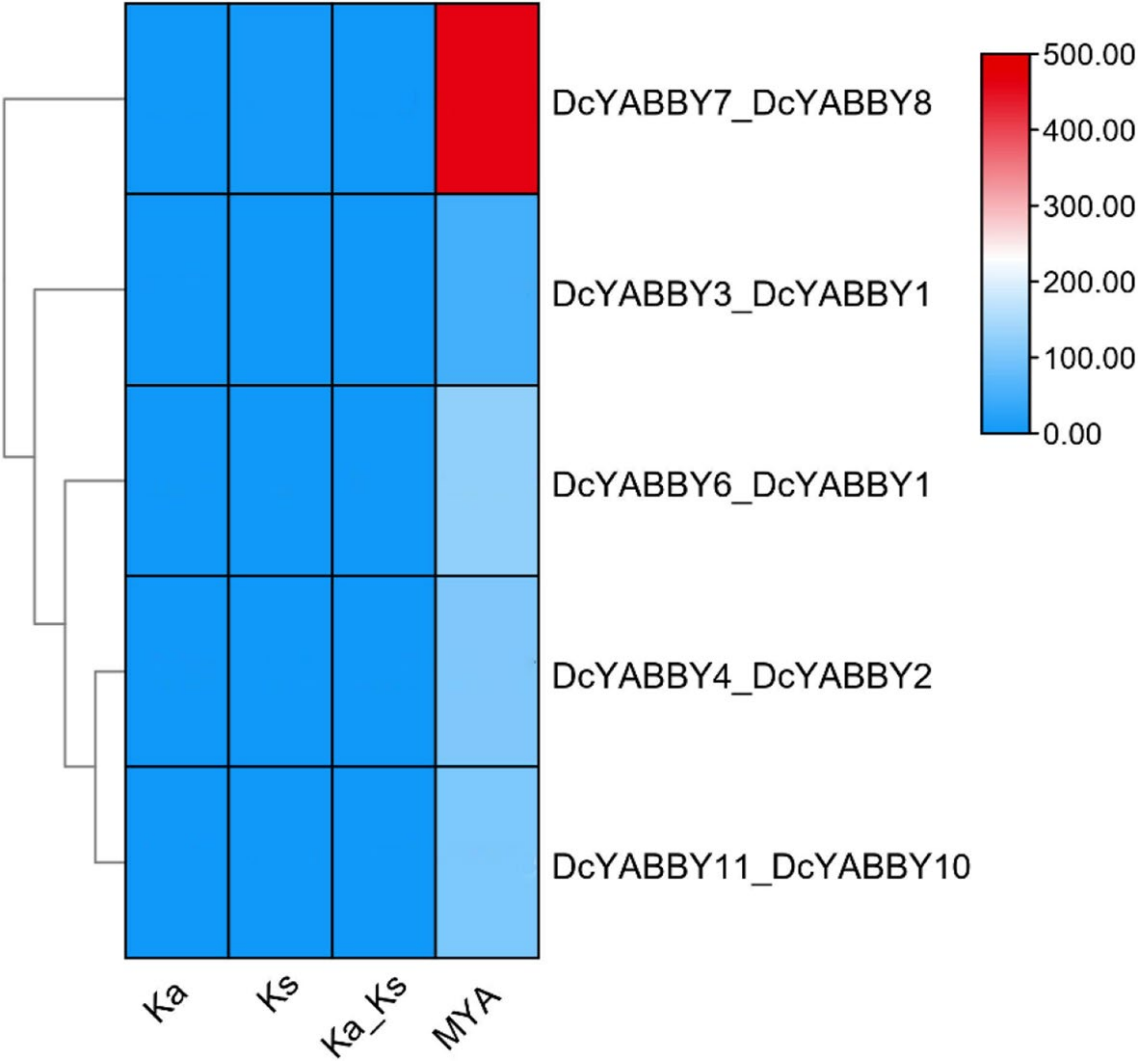
The representation of Ka/Ks the ratio of non-synonymous substitution (Ka) over synonymous substitution (Ks) mutations. The gene duplication over selection and evolutionary pressure to paralogous pairs of potato *StYAB* genes determined on the basis of Ks and Ka values

A phylogenetic relationship tree was made among *YABBY* genes of *D. carota, A. thaliana, C. sativus*, and *C. maxima*. *D. carota YABBY* genes are highlighted with a small red triangle symbol. The figure shows the division of 37 *YABBY* genes of four different crops. The grouping is based on the typical Arabidopsis phylogenetic grouping system. The results of phylogenetic analysis depicted that 11 *DcYABBY* proteins were distributed among 5 subgroups named *AtINO*, *AtCRC*, *AtYAB5*, *AtAFO/AtYAB*3 and *AtYAB2* (Fig. 4, Table S4). Group *AtINO* consists of total 6 *YABBY* proteins, including 1 from *Arabidopsis* i.e. *AtINO*, and the remaining is *DcYABBY9*, Cm*YABBY*9, Cm*YABBY*10, Cs*YABBY*4, and Cs*YABBY*8. *AtCRC* group consist of 7 *YABBY-*like proteins that are *AtCRC*, *DcYABBY10*, *DcYABBY11*, Cm*YABBY*12, Cm*YABBY*11, Cm*YABBY*7 and Cs*YABBY*3. The *AtYAB5* group contained 8 *YABBY* proteins of which 1 is of *Arabidopsis AtYAB5*, 2 of carrot *DcYABBY8*, *DcYABBY5*, 3 of cucumber Cm*YABBY*4, Cm*YABBY*5, Cm*YABBY*8 and 2 of muskmelon Cs*YABBY*5, Cs*YABBY*2. The *AtAFO* contained 12 *YABBY-*like proteins in which 2 are of *Arabidopsis AtAFO*, AtYAB3 while 5 are of carrot, *DcYABBY1*, *DcYABBY2, DcYABBY 3, DcYABBY4, DcYABBY6* and 3 of musk melon Cm*YABBY*1, Cm*YABBY*3, Cm*YABBY*7. The last group *AtYAB2*, had 5 *YABBY*-like proteins, 1 of them is of *Arabidopsis AtYAB2*, 1 from carrot *DcYABBY7*, 1 of cucumber Cs*YABBY*1, and 2 of musk melon Cm*YABBY*6, Cm*YABBY*13. The members of the same clade represent the same structure and function. Therefore, it has been concluded that sequences of structurally similar proteins have variable and spatio-temporal functional similarity (Fig. 5).

### Evaluation of gene duplication and gene mapping of carrot *YABBY* genes

**Fig. 6:**
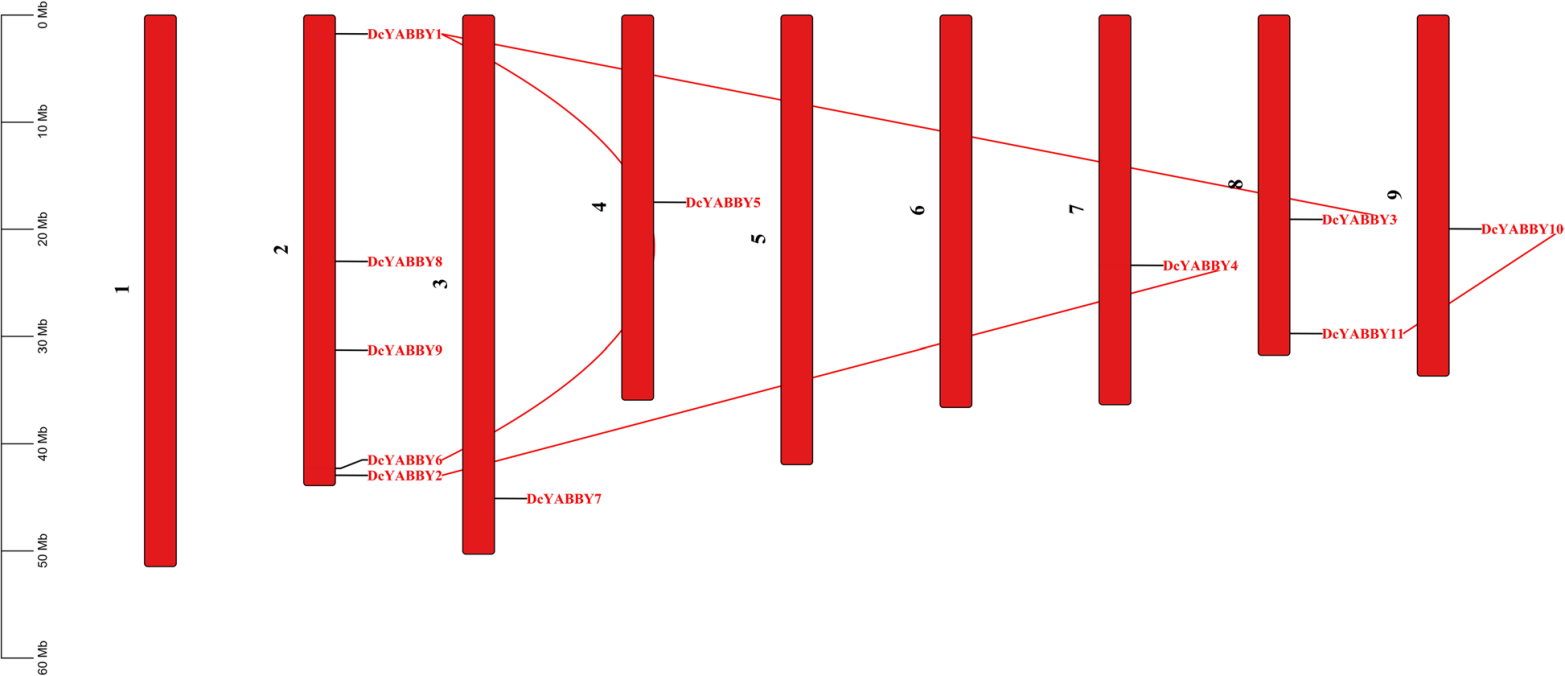
Chromosomal mapping showing the paralogues of *YABBY* genes with putative location. There 11 Y*ABBY* genes duplicated during the selection pressure and genomic rearrangement with retaining the ancestral function and gain of stable functional attributes in carrot genome

The duplication date of *DcYABBY* genes was calculated using the TB tool v1.098669 (Fig. 6). The Ka/Ks ratio extended from 0.08888631 in *DcYABBY7*_*DcYABBY8*, to 0.1821759 in *DcYABBY4*_*DcYABBY2* pair. The speculative date for segmental duplication date was calculated between 51.0916678 (Mya) for paralogous pair *DcYABBY3*_*DcYABBY1* as highest, to 463.797915 Mya for paralogous pair *DcYABBY7*_*DcYABBY8* as lowest. The Ka/Ks ratios of all the 5 paralogous group pairs were greater than 0.05 and less than 1 ultimatley resulting in a significant divergence during purifying selection period (Fig. 6).

### Analysis of *Cis-*regulatory elements

**Fig. 7:**
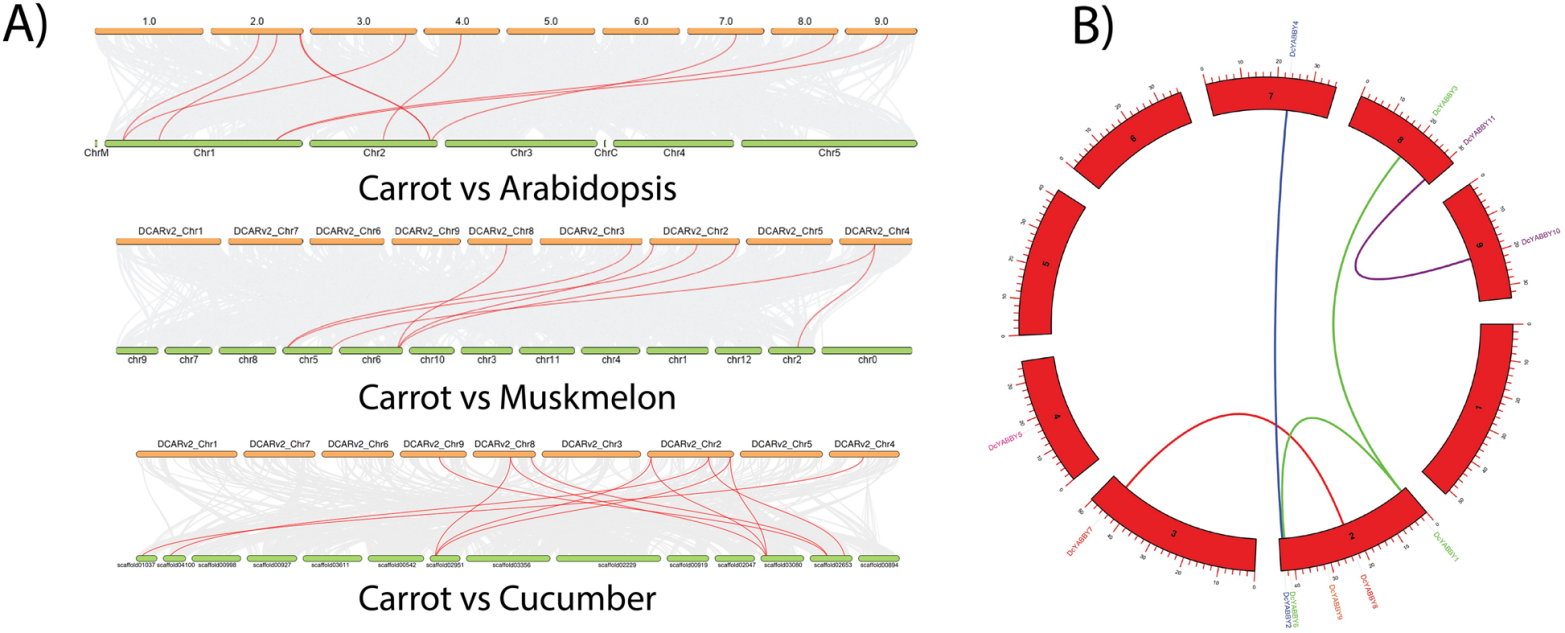
**A** Dual synteny analysis of Carrot-Arabidopsis, Carrot-Cucumber, and Carrot-Musk melon were performed to unmask the similarity and gene duplication distribution. Genomic regions of afore mentioned plants species have been shown with fine gene duplication and structural sharing among them.** B** Genome-wide synteny analysis of carrot *DcYABBY* genes showing paralogous gene pairs in the carrot genome

Various *Cis-*regulatory elements with different physiological and biological functions were observed. Many of these include light-responsive elements, specific responsive elements to abscisc acid, salicylic acid and gibberellins, anaerobic induction, meristem expression, seed-specific regulation, zien metabolism, and some defensive regulatory elements (Fig. 7, Table 2). Mainly, ARE element was present in 4 out of 11 *DcYABBY* genes that have a function in anaerobic induction, 5 *DcYABBY* genes contained box 4 element, which is a fragment of a conserved DNA module that helps in response to light, that takes part in light responsiveness, ABRE element was contained by 4 *DcYABBY* genes which have function related to abscisic acid response, 5 *DcYABBY* genes possessed TGACG element which is a sensitive element to methyl jasmonic acid, 1 *DcYABBY* gene possessed TCA element linked to respond with salicylic acid, 1 *DcYABBY* gene displayed wound-responsive WUN motif. While one *DcYABBY* gene indicated TC-rich repeats that have activity related to stress and defence, 2 *DcYABBY* genes contained CAT-box, which responds to meristemic expression, and MBS element was possessed by 3 *DcYABBY* genes which respond to drought induce ability, 2 *DcYABBY* genes have LTR element which is linked to respond in low-temperature, 1 *DcYABBY* gene have RY-element which is mainly associated with seed regulation. On the contrary, 1 *DcYABBY* gene contained GCN4_motif, which takes account of endosperm expression, and 2 *DcYABBY* genes possess AT-rich elements involved in DNA binding protein ATBP-1.

**Table 2:** The spatio-temporal functional distribution of *YABBY* gene’s *Cis-*regulatory elements among various tissues and organs during plant biological development process

Sr #	*Cis*-Elements	Function	References
1	ABRE	*cis*-acting element involved in the abscisc acid responsiveness	[50]
2	ARE	cis-acting regulatory element essential for the anaerobic induction	[51]
3	AT-rich element	binding site of AT-rich DNA binding protein (ATBP-1)	[51]
4	Box 4	part of a conserved DNA module involved in light responsiveness	[52]
5	CAAT-box	common cis-acting element in promoter and enhancer regions	[53]
6	CAT-box	*cis*-acting regulatory element related to meristem expression	[54]
7	CCAAT-box	MYBHv1 binding site	[55]
8	CGTCA-motif	cis-acting regulatory element involved in the MeJA-responsiveness	[56]
9	G-box	cis-acting regulatory element involved in light responsiveness	[57]
10	GT1-motif	light responsive element	[58]
11	GCN4_motif	cis-regulatory element involved in endosperm expression	[59]
12	LTR	cis-acting element involved in low-temperature responsiveness	[60]
13	MBS	MYB binding site involved in drought inducibility	[61]
14	MRE	MYB binding site involved in light responsiveness	[62]
15	MSA-like	cis-acting element involved in cell cycle regulation	[63]
16	O2-site	cis-acting regulatory element involved in zein metabolism regulation	[64]
17	P-box	gibberellin-responsive element	[65]
18	RY-element	cis-acting regulatory element involved in seed-specific regulation	[66]
19	TATA-box	core promoter element around − 30 of transcription start	[67]
20	TATC-box	cis-acting element involved in gibberellin-responsiveness	[68]
21	TCA-element	cis-acting element involved in salicylic acid responsiveness	[64]
22	TC-rich repeats	cis-acting element involved in defense and stress responsiveness	[64]
23	TCCC-motif	part of a light responsive element	[53]
24	TCT-motif	part of a light responsive element	[53]
25	TGACG-motif	cis-acting regulatory element involved in the MeJA-responsiveness	[53]

**Fig. 8:**
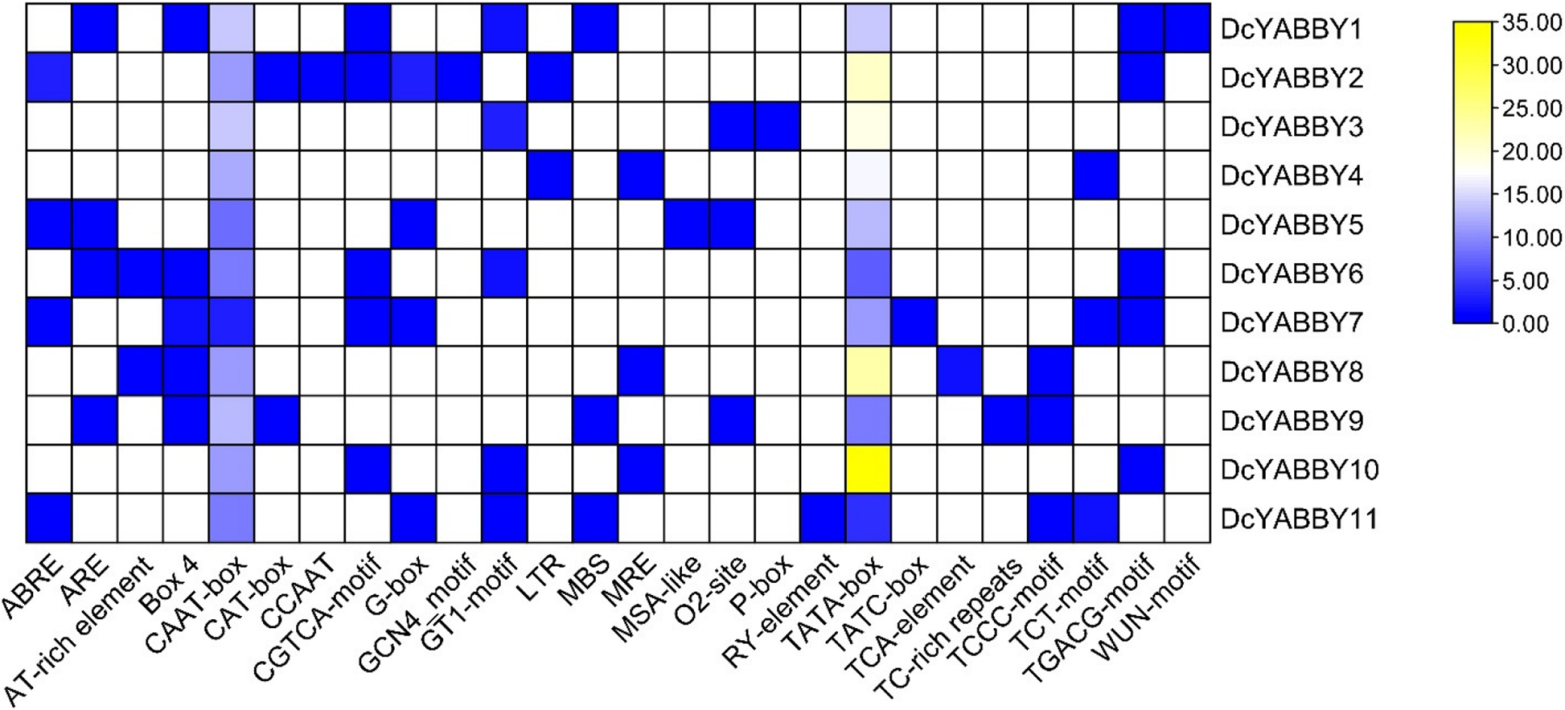
The graphical representation of *Cis*-regulatory elements of *DcYABBY* genes with intensity to their function at various levels via each gene’s promoter region. The functional intensity can be defined with red to blue colours from higher to low level during biochemical and physiological plant development respectively

The MSA-like element was expressed by 1 *DcYABBY* gene, which regulates the cell cycle. 1 *DcYABBY* gene contained CCAT, which is a common binding site for MYBHv1 while 5 *DcYABBY* genes showed CGTCA-motif that is also involved in methyl jasmonic acid responsiveness, 4 *DcYABBY* genes have G-box that helps in responding to light, 5 *DcYABBY* genes contained GT1-motif and 3 *DcYABBY* genes possessed MRE both of which are light-responsive element, O2-site was possessed by 3 *DcYABBY* genes which have a very important role in zien metabolism, p-box TATC box and TCA element are only contained by 1 *DcYABBY* gene and first two are gibberellin responsive elements and the last is salicylic acid responding element. TCCC-motif, TCT, and TGACG-motif contain 3 and 5 *DcYABBY* genes with varying functions (Figs. 7 and 8).

The physiological and biochemical functions with their orthologues in Arabidopsis of DcYABBY genes were studied with the help of gene ontology study (Table 3).

**Table 3:** *DcYABBY* genes have physiological and biochemical functions with their orthologues in Arabidopsis

Source ID	Gene ID	Gene ontology	*A. thaliana* Orthologues	Function	Reference
DCAR_004921	DcYABBY1	GO:0007275, GO:0032502	YABBY3, (AT4G00180)	Transcription cis-regulatory region & DNA-binding transcription factor activity, Ligand binding domain	[69]
DCAR_008543	DcYABBY2	GO:0007275, GO:0009909, GO:0009933, GO:0009944, GO:0010093, GO:0010154, GO:0010158, GO:0010450, GO:0032502, GO:0045165, GO:0090706, GO:1902183,	YABBY1, (AT2G45190)	Transcription cis-regulatory region & DNA-binding transcription factor activity, Ligand binding domain	[70]
DCAR_027801	DcYABBY3	GO:0007275, GO:0032502	YABBY3, (AT4G00180)	Transcription cis-regulatory region binding, DNA-binding transcription factor activity, Ligand binding domain	[71]
DCAR_031517	DcYABBY4	GO:0007275, GO:0032502	YABBY1, (AT2G45190)	Transcription cis-regulatory region & DNA-binding transcription factor activity, Ligand binding domain	[72]
DCAR_014892	DcYABBY5	GO:0007275, GO:0009944, GO:0032502, GO:1902183, GO:2000024	YABBY5, (AT2G26580)	Gene regulation	[73]
DCAR_008464	DcYABBY6	GO:0007275, GO:0032502	YABBY3, (AT4G00180)	Transcription cis-regulatory region & DNA-binding transcription factor activity, Ligand binding domain	[73]
DCAR_012254	DcYABBY7	GO:0007275, GO:0032502	YABBY2, (AT1G08465)	Transcription cis-regulatory region binding, DNA-binding transcription factor activity, Ligand binding domain	[74]
DCAR_006190	DcYABBY8	GO:0007275, GO:0032502	YABBY2, (AT1G08465)	Transcription cis-regulatory region binding, DNA-binding transcription factor activity, Ligand binding domain	[75]
DCAR_007074	DcYABBY9	GO:0007275, GO:0032502	INO, (AT1G23420)	DNA-binding transcription factor activity, Ligand binding domain	[76]
DCAR_030050	DcYABBY10	GO:0007275, GO:0032502	YABBY1, (AT2G45190)	Transcription cis-regulatory region binding, DNA-binding transcription factor activity, Ligand binding domain	[77]
DCAR_026683	DcYABBY11	GO:0007275, GO:0010254, GO:0010582, GO:0032502, GO:0048440, GO:0048479	CRC, (AT1G69180)	Transcription cis-regulatory region binding, DNA-binding transcription factor activity, Ligand binding domain	[78]

### Transcriptomic analysis of carrot *YABBY* genes

**Fig. 9:**
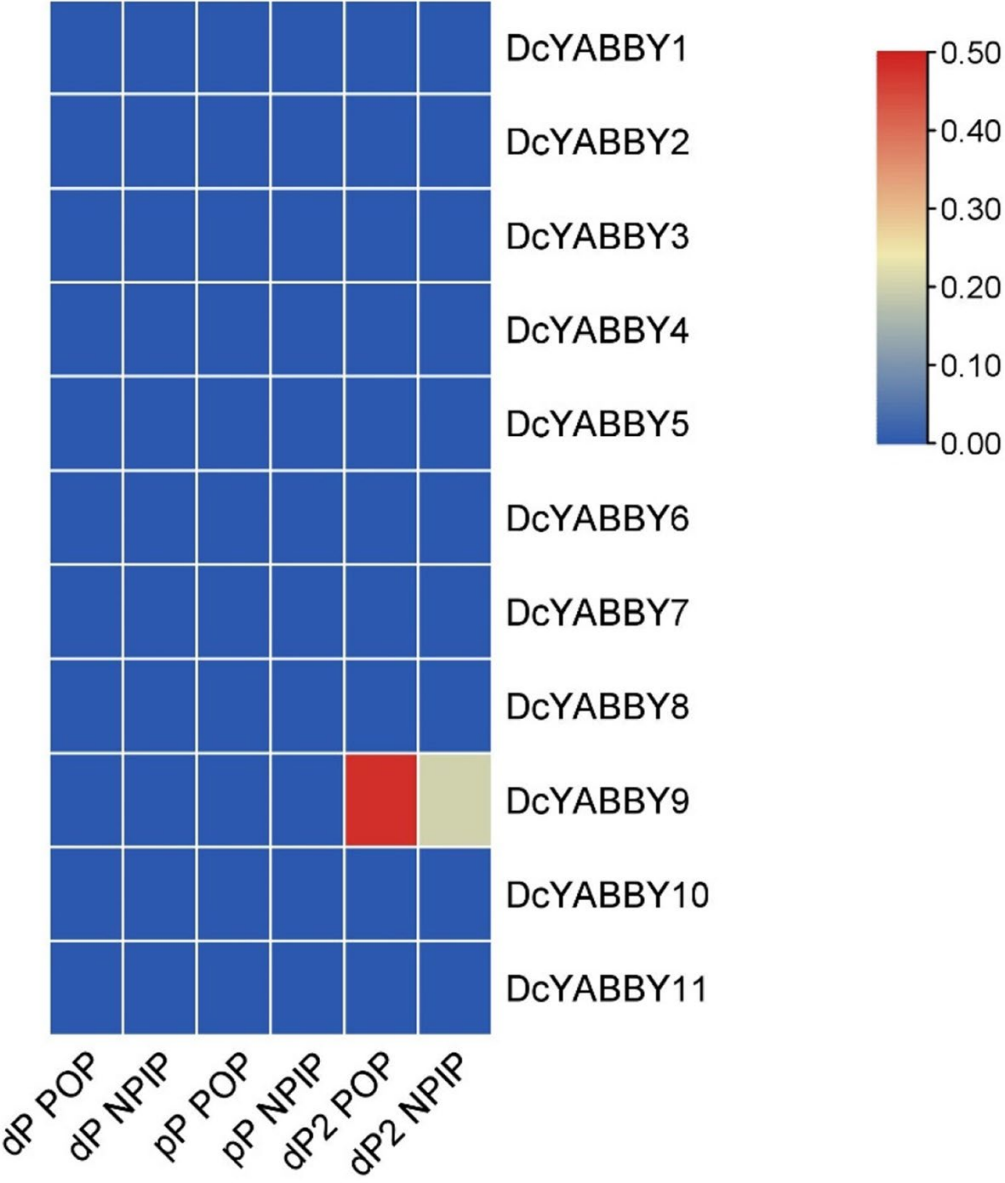
The Heat map of carrot *YABBY* genes responsible for pigmentation of anthocyanin are represented with higher intensity to low with red to blue colors. dP POP (Dark Purple Outer Phelom), dP NPIP (Dark Purple Inner), pP POP (Pale Purple Outer Phelom) and pP NPIP (Pale Purple Inner Phelom)

Regarding gene expression among all the 11 *DcYABBY* genes, only 1 has been involved in anthocyanin pigmentation in the carrot taproots. *DcYABBY9* (DCAR_007074) was expressed in dP2 POP and dP2 NPIP (Fig. 8). The extent of gene expression was slightly varied among these replicates. So it was concluded that *DcYABBY9* helps build a dark purple color in the outer phloem of carrot taproot by influencing more anthocyanin pigmentation [41, 42] (Fig. 9).

### Putative miRNA targets in carrot

Consequently, 5 miRNAs target the three genes i.e. *DcYABBY2*, *DcYABBY3* and *DcYABBY5* of the total 11 *DcYABBY* genes. *DcYABBY* 2 is the gene targeted by 3 mature miRNAs with different PmiREN IDs. On the other hand, *DcYABBY* 3 and 5 were targeted by 1 of the same mature miRNA (Table 4). None of the mature miRNAs targeted the remaining 8 DcYABBY genes. So, this indicated that *DcYABBY* 2 was the individual gene targeted by the maximum number of mature miRNAs. While discussing based on groups, *AtAFO* was targeted by 4 mature miRNAs. In contrast, the minimum number of miRNA targeted groups was *AtYAB5*, which was targeted by only 1 miRNA (Table 5).

**Table 4:** Representation of miRNAs with their targeting genes, length, starts and aligned sequence details

miRNA ID	Target ID	Length	Start-end	miRNA aligned fragment
Dca-MIR408a	DcYABBY5	21	1–21	UGCACUGCCUCUUCCCUGGCU
Dca-MIR168a	DcYABBY2	22	1–22	UCGCUUGGUGCAGGUCGGGACC
Dca-MIR168b	DcYABBY2	22	1–22	UCGCUUGGUGCAGGUCGGGACC
Dca-MIR168c	DcYABBY2	22	1–22	UCGCUUGGUGCAGGUCGGGACC
Dca-MIR408a	DcYABBY3	21	1–21	UGCACUGCCUCUUCCCUGGCU

**Table 5:** Functions of miRNAs and their role in gene regulation during the developmental stages

miRNA ID	Target gene ID	Function	Reference
Dca-MIR408a	DcYABBY5	Peptide chain release factor, Plantacyanin, Heat Regulation	[79, 80]
Dca-MIR168a	DcYABBY2	Regulates AGO1 for gene silencing, Response to Bacterial Infection	[79, 81]
Dca-MIR168b	DcYABBY2	Regulates AGO1 for gene silencing, Response to Bacterial Infection	[79, 81]
Dca-MIR168c	DcYABBY2	Regulates AGO1 for gene silencing, Response to Bacterial Infection	[79, 81]
Dca-MIR408a	DcYABBY3	Peptide chain release factor, Plantacyanin, Heat Regulation	[79, 80]

## Discussion

Plant specific Transcription factors (PSTrFs) are important molecules with spatio-temporal function and support during plant development and growth. PSTrFs are key in defining the fate of strong biological development and biochemical actions^22^. *YABBY* genes in carrots and other species act as TrFs and provide basic support during the developmental cycle. Phylogenetic and conserved sequences analysis of *YABBY* TrFs in *Arabidopsis thaliana* and eggplant of span into five families, including *AtINO*, *AtCRC*, *AtYAB5*, *AtAFO*/*AtYAB*3, *AtYAB2.* The genomic identification of *DcYABBY* genes has been completed by comparing recently released genomic features from comprehensive plant repository Ensembl plants [26, 27], [82] (Table 1). Phylogenic findings characterize 11 *YABBY* genes of *A. thaliana* into five groups *AtINO*, *AtCRC*, *AtYAB5*, *AtAFO*/*AtYAB*3, and *AtYAB2* (Fig. 5, Table S4). The following distribution leads to new insights into less sequence-level conservation for YABBY carrot genes. The number of *YABBY* TFs in the carrot is less than other domestic and model plant i.e. rice possesses 30 *OsYABBY,* Arabidopsis; 36 *AtYABBY*, tomato; 34 *SiYABBY* [83] banana; 74 *MaYABBY* and [84] Chinese cabbage; 76 *BrATYABBY* [85, 86].

The less correlated number of introns and exons in these families depicts the purifying selection and evolutionary instability with divergent evolution. Higher introns in the plant genome provide information regarding its evolutionary and genomic stability. The genomic architecture and correlation in phylogeny depicted a clear picture of evolutionary correlation among various *YABBY* gene families [87, 88].

The genomic feature of similar characters possessing genes had the same number of introns and exons at genomic level (Table S2). Same clades of *DcYABBY* have an almost similar number of exons and introns (Fig. 2) while various clades of different families have different number of introns and exons i.e. Arabidopsis, rice and soybean, suggesting conservation of characteristic sequences among them [89, 90].

The conservation of sequence to function level has been assessed by identification of motif (Fig. 3) sequence among all *DcYABBY* genes at protein level spanning from 15 to 167 bp (Table S3) amino acids along with frequently existing HMG box domain (Table S5, S9). All members of *DcYABBY* proteins comprised Motif 1 and motifs named Hmg_box and Hmg_box2 are also residing, and at a functional level, HMG box is responsible for binding the DNA. The sequence-level investigations correspond to similarities at the sequence level, leading to functional and structural correlation. The preservation of evolutionary traits leads to the rearrangement and structuring of domains while maintaining consistent functionality. Confirming these functional similarities, gene ontology (GO) annotation of *AtAFO* genes in *Arabidopsis thaliana* has been undertaken. Evolutionary gene expansion might cause arrangements of the *YABBY* domains to have similar motif patterns in different groups. To recognize the possible function of the Group *AtAFO*, which contained five *DcYABBY* genes and several similar motifs, GO annotations of the Group *AtAFO* genes in Arabidopsis resulting in similarities among *DcYABBY* genes and *AtAFO* with transcriptional functions, cis-regulatory region binding, DNA-binding, protein binding and ion channelling (Table 3) [91, 92]. The structural arrangement of the *DcYABBY* genes was conserved among all the five divided groups of species i.e. Arabidopsis, Cucumber, and Musk melon [93]. Furthermore, an investigation of subcellular localization among *DcYABBY* proteins using the online web tool WoLF PSORT [34] has been performed and resulted in nuclear localization of *DcYABBY* proteins to cytoplasm and chloroplast while these all were commonly present in the nucleus (Table S1). Segmental and tandem duplication was observed in the *YABBY* gene family at various chromosomes, which is a clear picture of genomic rearrangements during the evolutionary process. These rearrangements at the genome level lead to the development of new characters, i.e., conservative sequences and domains for sustaining the functional characteristics of plants [94]. The best-known tandem and segmental duplication in carrot *YABBY* genes on chromosome 2 (Fig. 7A) and *DcYABBY1* with *DcYABBY3*, *DcYABBY2* with *DcYABBY4* and *DcYABBY10* with *DcYABBY11* (Fig. 7B) have been found in this research. Segmental duplications are dominant in chickpea [93] pigeon pea [15, 92], and in the *YABBY* gene family. These results indicate the main process of gene and conserved region expansion at the genomic level due to duplications of *YABBY* genes throughout the evolution of eukaryotic plants [95, 96]. The purifying and evolutionary selection at amino acid level and substitution ratio i.e. Ka and Ks (Fig. 5) support these findings that YABBY genes have evolved and retain their function through evolution. Ka/Ks < 1 ratio leads to purifying selection, and positive selection pressure leads to Ka/Ks > 1 values. This selection pressure by the biological clock and environment leads to the rearrangement of specific blocks and domains at the level, resulting in the origination of new characteristics across the species [97]. In current investigations, variation among ratios of Ka /Ks between *DcYABBY* genes is less and predicted values of Ka/Ks ranges from 0.09 to 0.29 which are less than 1. The aforementioned results showing that sequences of *YABBY* in all families underwent purifying selection pressure and can only affect few sites during the process of evolution (Fig. 5). The expression profile of DcYABBY genes in several carrot experiments using available RNA sequencing data was analysed, resulting in the conclusion that anthocyanin can accumulate in purple-rooted carrots. Genomic diversity indicates anthocyanin expression either in taproot and tissue specificity confined to phloem’s root or xylem’s tissues. Insilco information and computation i.e. linkage mapping and transcriptomic analysis have been used to assess the hidden facts about anthocyanin pigmentation in inner and outer phloem of carrot taproots in two different genomic backgrounds [41, 42]. Cluster analysis and gene omnibus at NCBI were used to unhide the spatio temporal function of carrot *YABBY* genes and 1 out of 11 *DcYABBY* genes involved in anthocyanin pigmentation in the carrot taproot [41, 42]. *DcYABBY9* (DCAR_007074) was highly expressed in dP2 POP and dP2 NPIP (Fig. 9) [98]. Except *for DcYABBY9, all other genes have no expression or* function related to anthocyanin pigmentation. The cis-regulatory analysis also predicts that *DcYABBY9* also has a role in light responsiveness, zein metabolism regulation, regulatory function related to meristem expression, involved in drought-induce ability, essential for the anaerobic metabolism during abiotic stress and defence responsiveness (Fig. 8, Table 2). The orthologue of *DcYABBY9* in Carrot is AT1G23420 and *AtINO*, which are in the same group and have a role in DNA and metal ion binding. The orthologues of these three aforementioned Arabidopsis proteins are *DcYABBY* 10, 11 and 12 which can lead to conclude their similar functions in the Carrot plant as of its orthologues in Arabidopsis*.*

MicroRNAs are important in plant growth regulation processes extending from developmental to defending against pathogens and sustaining internal immunity [99–102]. MiRNAs are present in most plant species in a conserved manner with specified functions. Most of the *DcYABBY* genes have transcriptional-associated functions, resulting in the suppression of activity to miRNAs. It is the only reason that three out of 11 *DcYABBY* genes were targeted by MIR408 and MIR168 family members (Table 5). MIR408 targeted two *DcYABBY* genes while MIR168 to one gene. These two micro RNAs targeted *DcYABBY2*, *DcYABBY3,* and *DcYABBY5*, respectively. *DcYABBY2* was targeted by three miRNAs i.e. MIR168a and MIR168b, which reside on chromosome 1 and MIR168c at chromosome 9 of carrot. Meanwhile *DcYABBY3* and *DcYABBY5* were both targeted by MIR408a located on chromosome 1. This scenario provides a basis for the conclusion that most of their origin and activity are driven by chromosome 1. MiR408 is abundantly present in different plant species that specifically hits mRNAs related to copper-binding protein. Overexpression of *MIR408* was shown to improve phenotypic properties of *Arabidopsis* by increasing leaf area, plant height, petiole length, flower size, and silique length, which ultimately enhances seed yield and biomass [103]. MiR408 has diverse roles in Arabidopsis, from which we can assume that this micro RNA targeting *DcYABBY* genes can also play an important role in enriching carrot nutrients. Overexpression of miR408 triggered enhanced drought tolerance in chickpeas by causing plantacyanin transcript suppression, which regulates DREB and other genes related to drought response [104]. In response to *miR168*, Argonaute (AGO1) is upregulated, activating the RNA silencing complex (RISC) in tomatoes to modulate the small RNA regulatory pathway [105]. The suppression of miR168 by a target mimic (MIM168) not only improves grain yield and shortens rice flowering time but enhances immunity to Magnaporthe oryzae, the causal agent of rice blast disease.

## Conclusion

This study comprehensively analyzed *DcYABBY* PSTrFs genes in the carrot genome. The 11 *DcYABBY* genes were classified into five groups, and some of the structural and functional properties of each *DcYABBY* member were characterized. Some of the *DcYABBY* genes were involved in taproot pigmentation. MiRNA data targeting *the DcYABBY* gene in anthocyanin pigmentation development in carrot suggest their role in growth and development. The in-depth computational analysis of carrot *YABBY* proteins revealed in the current study is the first step to undermining the hidden realities of *YABBY* proteins in carrots and in contrast to other crops. Complex interaction and cooperation at the functional level of *YABBY* proteins portray their expression level and interaction with different transcription factors. The presence of an almost similar number of *YABBY* genes i.e. 33 in (tomato), 34 (pepper), and 35 (potato), and a relatively higher number in other plants 78 in soybean and 51 in carrot suggested the variation in *YABBY* genes at a genomic, structural and functional level.

### Supplementary Information

**Supplementary Material 1.**

## Data Availability

The datasets analyzed during the current study are available in the Ensemble plants database (https://plants.ensembl.org/index.html), NCBI-GEO database (https://www.ncbi.nlm.nih.gov/geo/query/acc.cgi?acc=GSE181611), and miRBase database (https://mirbase.org/) repository. All data collected or generated has ben provided in manuscript and its supplementary file. Carrot YABBY genes source accession numbers along with their repository web links.

**Table:** 

YABBY gene	Source Accession	Gene Repository Web links
Name	No.	
DcYABBY1	DCAR_004921	https://plants.ensembl.org/Daucus_carota/Gene/Summary?g=DCAR_004921;r=2:1743358-1745477;t=KZN04084;db=core
DcYABBY2	DCAR_008543	https://plants.ensembl.org/Daucus_carota/Gene/Summary?g=DCAR_008543;r=2:42935114-42937362;t=KZN07706;db=core
DcYABBY3	DCAR_027801	https://plants.ensembl.org/Daucus_carota/Gene/Summary?g=DCAR_027801;r=8:19069882-19071293;t=KZM84777;db=core
DcYABBY4	DCAR_031517	https://plants.ensembl.org/Daucus_carota/Gene/Summary?g=DCAR_031517;r=7:23365142-23367453;t=KZM88020;db=core
DcYABBY5	DCAR_014892	https://plants.ensembl.org/Daucus_carota/Gene/Summary?g=DCAR_014892;r=4:17475005-17477702;t=KZM97746;db=core
DcYABBY6	DCAR_008464	https://plants.ensembl.org/Daucus_carota/Gene/Summary?g=DCAR_008464;r=2:42290093-42292399;t=KZN07627;db=core
DcYABBY7	DCAR_012254	https://plants.ensembl.org/Daucus_carota/Gene/Summary?g=DCAR_012254;r=3:45104800-45108502;t=KZN03498;db=core
DcYABBY8	DCAR_006190	https://plants.ensembl.org/Daucus_carota/Gene/Summary?g=DCAR_006190;r=2:22992937-22996396;t=KZN05353;db=core
DcYABBY9	DCAR_007074	https://plants.ensembl.org/Daucus_carota/Gene/Summary?g=DCAR_007074;r=2:31254231-31255572;t=KZN06237;db=core
DcYABBY10	DCAR_030050	https://plants.ensembl.org/Daucus_carota/Gene/Summary?g=DCAR_030050;r=9:19957276-19958590;t=KZM82481;db=core
DcYABBY11	DCAR_026683	https://plants.ensembl.org/Daucus_carota/Gene/Summary?g=DCAR_026683;r=8:29737387-29738680;t=KZM85895;db=core Carrot YABBY genes source accession numbers along with their repository web links.

**Table:** 

miRNA ID	Target ID	miRNA Repository Web links
Dca-MIR408a	DcYABBY5	https://pmiren.com/singlemirna?Accession=PmiREN034271
Dca-MIR168a	DcYABBY2	https://pmiren.com/singlemirna?Accession=PmiREN034231
Dca-MIR168b	DcYABBY2	https://pmiren.com/singlemirna?Accession=PmiREN034232
Dca-MIR168c	DcYABBY2	https://pmiren.com/singlemirna?Accession=PmiREN034233
Dca-MIR408a	DcYABBY3	https://pmiren.com/singlemirna?Accession=PmiREN034271
