# Supplementary material for: Genome-wide analysis of plant specific YABBY transcription factor gene family in carrot (Dacus carota) and its comparison with Arabidopsis
Source: BMC Genom Data. 2024 Mar 5;25:26. doi: 10.1186/s12863-024-01210-4 (PMC10916311; doi:10.1186/s12863-024-01210-4)
Supplement: Supplementary file 1 — Supplementary Material 1. [file 12863_2024_1210_MOESM1_ESM.docx]

1. **Supplementary Material:**

**Tables:**

**Table S1:** Table represents Subcellular Localization of 11 eleven YABBY genes present in Carrot.

| Gene IDs | Nucleus | Cytoplasm | Chloroplast | Cytoplasm + Nucleus |
| --- | --- | --- | --- | --- |
| DcYABBY1 | 10.5 |  | 3 | 6 |
| DcYABBY2 | 12 |  | 2 |  |
| DcYABBY3 | 9 |  | 5 |  |
| DcYABBY4 | 13 | 1 |  |  |
| DcYABBY5 | 9 |  | 2 |  |
| DcYABBY6 | 12 |  | 7 |  |
| DcYABBY7 | 7 |  | 7 |  |
| DcYABBY8 | 7 | 4 | 3 |  |
| DcYABBY9 | 13 | 1 |  |  |
| DcYABBY10 | 13 | 1 |  |  |
| DcYABBY11 | 14 |  |  |  |

**Table S2**: Explaining Insilco predicted number of introns and exons of carrot *YABBY* genes.

| Gene Name | Source Accession Number | Number of Introns | Number of Exons |
| --- | --- | --- | --- |
| DcYABBY1 | DCAR_004921 | 6 | 7 |
| DcYABBY2 | DCAR_008543 | 6 | 7 |
| DcYABBY3 | DCAR_027801 | 6 | 7 |
| DcYABBY4 | DCAR_031517 | 6 | 7 |
| DcYABBY5 | DCAR_014892 | 6 | 7 |
| DcYABBY6 | DCAR_008464 | 6 | 7 |
| DcYABBY7 | DCAR_012254 | 4 | 5 |
| DcYABBY8 | DCAR_006190 | 3 | 4 |
| DcYABBY9 | DCAR_007074 | 6 | 7 |
| DcYABBY10 | DCAR_030050 | 5 | 6 |
| DcYABBY11 | DCAR_026683 | 6 | 7 |

**Table S3:** Represents distributions and occurrence of motifs in all the eleven predicted *DcYABBY* genes.

| Genes | Motifs | | | | | | | | | | | | | | | | | | | |
| --- | --- | --- | --- | --- | --- | --- | --- | --- | --- | --- | --- | --- | --- | --- | --- | --- | --- | --- | --- | --- |
| DcYABBY3 | 2 | 5 | 14 | 4 | YABBY | 6 | 3 |  |  |  |  |  |  |  |  |  |  |  |  |  |
| DcYABBY1 | 2 | 5 | 14 | 4 | YABBY | 6 | 3 |  |  |  |  |  |  |  |  |  |  |  |  |  |
| DcYABBY6 | 2 |  | 14 | 4 | YABBY | 6 |  | 19 | 13 |  |  |  |  |  |  | 15 |  |  |  |  |
| DcYABBY2 | 2 |  |  | 4 | YABBY |  | 3 |  |  | 7 | 8 |  |  |  |  |  |  |  |  |  |
| DcYABBY4 | 2 |  |  | 4 | YABBY |  |  |  |  |  | 8 |  |  |  |  |  |  |  |  |  |
| DcYABBY5 | 2 |  |  |  | YABBY | 6 |  |  |  | 7 |  | 20 |  |  |  |  |  |  |  |  |
| DcYABBY9 |  |  |  |  | YABBY |  |  |  |  |  |  | 20 | 18 | 9 |  |  |  |  |  |  |
| DcYABBY7 |  |  |  | 4 | YABBY |  |  | 19 | 13 |  |  |  |  | 9 | 12 | 15 | 11 |  |  |  |
| DcYABBY8 | 2 |  |  |  | YABBY |  |  |  |  |  |  |  |  |  |  |  | 11 | 17 |  |  |
| DcYABBY10 | 2 |  |  |  | YABBY |  |  |  |  |  |  |  |  |  | 12 |  |  |  | 16 | 10 |
| DcYABBY11 | 2 |  |  |  | YABBY |  |  |  |  |  |  |  | 18 |  |  |  |  | 17 | 16 | 10 |

| Groups | Total genes | *Duacus carrota*  genes | Gene IDs | *Cucumis sativus* genes | Gene IDs | *Cucumis melo*  genes | Gene IDs | *Arabidopsis thaliana*  genes | Gene IDs |
| --- | --- | --- | --- | --- | --- | --- | --- | --- | --- |
| AtCRC | 7 | 2 | DcYABBY10, DcYABBY11 | 1 | CsYABBY3 | 3 | CmYABBY11, CmYABBY12, CmYABBY7 | 1 | AtCRC |
| AtINO | 6 | 1 | DcYABBY9 | 2 | CsYABBY8, CsYABBY4 | 2 | CmYABBY9, CmYABBY10 | 1 | AtINO |
| AtYAB2 | 5 | 1 | DcYABBY7 | 1 | CsYABBY1 | 2 | CmYABBY6, CmYABBY13 | 1 | AtYAB2 |
| AtAFO  YAB3 | 12 | 5 | DcYABBY1, DcYABBY2, DcYABBY3, DcYABBY4, DcYABBY6 | 2 | CsYABBY6, CsYABBY7 | 3 | CmYABBY1, CmYABBY2, CmYABBY3 | 2 | AtAFO, AtYAB3 |
| AtYAB5 | 8 | 2 | DcYABBY5, DcYABBY8 | 2 | CsYABBY5, CsYABBY2 | 3 | CmYABBY4, CmYABBY5, CmYABBY8 | 1 | AtYAB5 |

**Table S4:** Explanation of carrot *YABBY* gene family members distribution among different groups based on phylogenetic analysis with Arabidopsis with other phylogenic members.

**Table S5:** The table represents total no. of motifs found in eleven *DcYABBY* genes along with their name, motif sequence and amino acid length.

| Sr # | Motif Name | Motif Sequence | AA Length |
| --- | --- | --- | --- |
| 1 | YABBY | LEIRIPCKRMLETVTVKCGQCCNLSYLTAVPPP  PVQSPSLDSSATLQSSLCSDSKRLQKKKKNKQ  LHCSSPKHESSSSSSNAPFVVKPPERKHRPPSA  YNRFMREEIQRIKAANPNIPHRDAFSAASKN  WARCSSITNMPLFTTISESSNNVCTKE | 167 |
| 2 | HMG_Box | DAFSAASKNWARCSSITNMPLFTTISESSNNVCTKE | 38 |
| 3 | HMG_Box 2 | NIPHRDAFSAASKNWARCSSITNMPLFTTISESSNNVCTKE | 45 |
| 4 | Ccdc124 | KAANPNIPHRDAFSAASKNWARCSSITNMPLFTTISESSNNVCTKE | 50 |
| 5 | Zinc_ribbon_12 | CKRMLETVTVKCGQCCNLSYLTAVPPPPVQSPS | 36 |
| 6 | Elf_1 | PCKRMLETVTVKCGQCCNLSYLTAVPPPPVQSPSLDSSATL | 41 |
| 7 | NADHdh_A3 | KPPERKHRPPSAYNRFMREEIQRIKAANPNI | 31 |
| 8 | Mu-like_com | TAVPPPPVQSPS | 12 |
| 9 | ZInc_ribbon_5 | LSYLTAVPPPPVQSP | 15 |
| 10 | DUF4770 | QSSLCSDSKRLQKKKKNKQLHCSSPKHESSSSSSNAPFVVKPP  ERKHRPPSAYNRFMREEIQRIKAANPNIPHRDAF | 77 |
